# Curcumin alleviates postprandial glycaemic response in healthy subjects: A cross-over, randomized controlled study

**DOI:** 10.1038/s41598-018-32032-x

**Published:** 2018-09-12

**Authors:** Rohith N. Thota, Cintia B. Dias, Kylie A. Abbott, Shamasunder H. Acharya, Manohar L. Garg

**Affiliations:** 1 Nutraceuticals Research Program, School of Biomedical Sciences & Pharmacy, University of Newcastle, Callaghan, NSW Australia; 2 Riddet Institute, Massey University, Palmerston North, New Zealand; 3 Department of Diabetes, John Hunter Hospital, Hunter New England Local Health District, New Lambton Heights, NSW Australia

## Abstract

In the current study, we aimed to evaluate the effects of a single dose of curcumin and/or fish oil on postprandial glycaemic parameters in healthy individuals. This was a randomised, placebo-controlled and crossover study. Sixteen (n = 16) volunteers were randomised to receive placebo, curcumin (180 mg) tablets, fish oil (1.2 g long chain omega-3 polyunsaturated fatty acids) capsules and curcumin + fish oil prior to a standard meal on 4 test days separated by a week. Blood glucose, serum insulin and triglycerides were measured at intervals between 0–120 min. Difference between the treatments was measured using two-way repeated measures analysis of variance and pair-wise comparisons using Wilcoxon signed-rank or paired t-test as appropriate. Postprandial glucose concentrations were significantly lower in the curcumin (60.6%, P = 0.0007) and curcumin + fishoil group (51%, P = 0.002) groups at 60 min from baseline. Compared with placebo, area under the curve (AUC) for change in blood glucose concentration was reduced by curcumin (36%, P = 0.003) and curcumin + fishoil (30%, 0.004), but not fish oil alone (p = 0.105). Both curcumin (P = 0.01) and curcumin + fishoil (P = 0.03) treatments significantly lowered postprandial insulin (AUC) by 26% in comparison with placebo. Curcumin, but not fish oil, reduces postprandial glycaemic response and insulin demand for glucose control.

## Introduction

Chronic elevation in postprandial blood glucose (PBG) and concomitant increase in insulin concentrations by calorie-dense foods has detrimental effects on metabolic regulation in many insulin sensitive tissues such as liver, pancreas and muscle in healthy individuals^1^. It has been hypothesised that sustained concentrations of high PBG increase oxidative stress and activation of pro-inflammatory pathways, eventually leading to the development of chronic metabolic diseases such as diabetes and cardiovascular disease^2^. In recent years, interest in the relationship between diet and health has promoted the use of nutrients or dietary components (such as fibre, resistant starch) for lowering the PBG^3,4^. Currently these dietary interventions are being subjected to rigorous scientific evaluation for their beneficial effects in either preventing or delaying the onset of the metabolic diseases.

Curcumin, a dietary bio-active derived from turmeric, has been shown to alleviate insulin resistance and lower fasting glucose in preclinical studies^5–7^. These effects were hypothesised to be mediated via lowering of low grade inflammation via down-regulation of nuclear factor kappa-light-chain-enhancer of activated B cells (NF-kB)^8^, cytoprotection of pancreatic β-cells by increasing the concentrations of anti-oxidant enzymes^9^ and via increasing expression of 5′ adenosine monophosphate-activated protein kinase (AMPK)^10^, a key regulator of glucose and lipid homeostasis. Clinical trials with curcumin supplementation reported positive^11^ and no effect^12^ on fasting glycaemic parameters in high risk and individuals with type 2 diabetes^13–15^. In addition, *In-vitro* studies demonstrated potent inhibitory effects of curcuminoids and its synthetic analogues on α-glucosidase enzyme and α-amylase, the key regulators of carbohydrate digestion^16,17^ linking curcumin and curcuminoids to PBG control.

Short term exposure of pancreatic β-cells to fatty acids has been shown to induce insulin secretion^18^. *In-vitro* studies have reported that glucose stimulated insulin secretion is greatly influenced with the degree of saturation of the fatty acids^19^. Long term exposure to poly- and mono- unsaturated fatty acids have shown cytoprotective effects on pancreatic β-cells, in contrast to the deleterious effects of long-chain saturated fatty acids^20,21^. Substituting dietary saturated fat with long chain omega-3 polyunsaturated fatty acids (LCn-3PUFA) in meals has shown improvements in the insulin sensitivity in individuals with and without type 2 diabetes^22–24^. However, results from multiple studies with long-term supplementation of LCn-3PUFA for improving insulin resistance are ambiguous^25^.

Despite availability of large body of evidence on physiological effects of curcumin and LCn-3PUFA on fasting parameters, a clear mechanism is lacking to explain their clinical effects. Moreover, the effects of curcumin were not previously explored in terms of post-prandial glycaemic regulation. As both curcumin and LCn-3PUFA are derived from diet or advised to be taken with meals (if supplemented), we hypothesised that long term beneficial effects of these two-dietary bio-actives could possibly achieved through post-meal control of glycaemic parameters. In the current randomised cross over trial, we aimed to evaluate effects of single dose of curcumin with or without LCn-3PUFA on glycaemic parameters (postprandial glucose and insulin) in response to a standardised meal rich in high carbohydrates and fat.

## Results

### Participant characteristics

**Figure 1:**
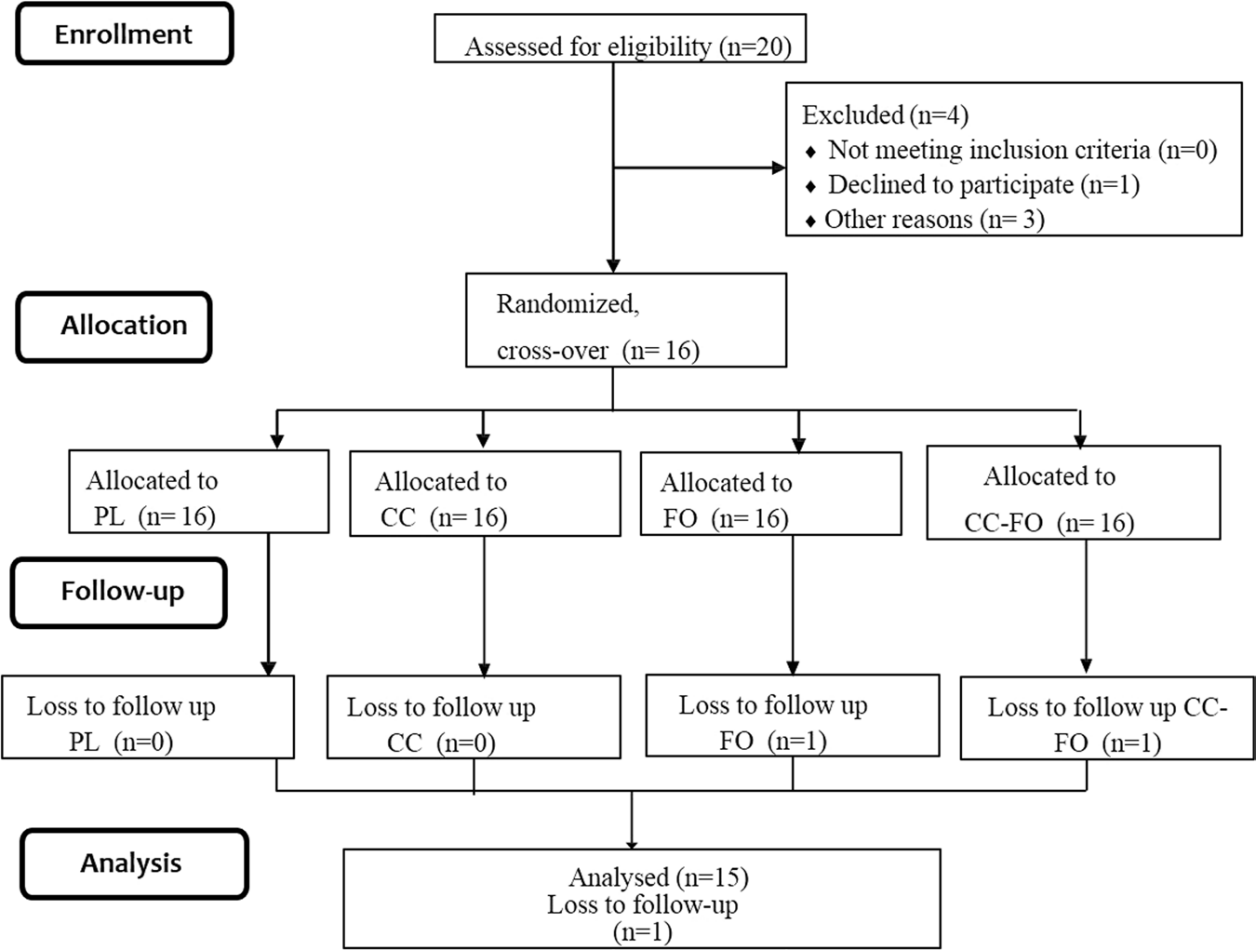
CONSORT flow chart – trial of study protocol.

Fifteen participants (8 males and 7 females) aged 26.3 ± 5.04 years with body mass index (BMI) of 24.9 ± 4.17 kg/m^2^ (Table 1) completed the study (Fig. 1). One female participant failed to follow-up after two visit days because of diagnosis with infection and is excluded from the analysis. Insulin value for one participant on single test day (allocated to fish oil alone) was excluded because of the unreliable data from the reanalysis of sample by pathology (Fig. 1).

**Table 1:** Baseline characteristics of the participants.

Variables	All participants (n = 15)
Age (years)	26.3 ± 5.04
Males/Females	8/7
Weight (kg)	71.1 ± 15.03
Muscle mass (kg)	28.1 ± 6.04
Body fat mass (kg)	20.8 ± 9.09
Fat free mass (kg)	49.2 ± 10.98
Body mass index (kg/m^2^)	24.9 ± 4.17
Percentage body fat	28.4 ± 8.98
Waist hip ratio	0.8 ± 0.05
Basal metabolic rate	1457 ± 216.1
Fasting Glucose (mmol/L)	5.4 ± 0.37
Fasting Insulin (mU/L)	8.0 ± 2.42
Fasting Triglycerides (mmol/L)	0.8 ± 0.26
Physical activity (metabolic equivalents-minutes/week)	678 (462, 1986)

Data are reported as means ± SD or median (25^th^ and 75^th^ percentile) as appropriate. SD-standard deviation. Physical activity levels of the participants were measured using standard International physical activity questionnaire – short form.

### Physical activity and dietary intake

Physical activity analysis indicated seven participants with low physical activity, five with moderate and three with high physical activity concentrations. There were no significant differences in energy, carbohydrate, sugar, starch, protein, total fat, polyunsaturated and monounsaturated fat and fibre intake between 24-h period preceding each of the four test days (Table 2).

**Table 2:** Composition of the habitual diets as consumed 24 h before the PL, FO, and CC and CC-FO test days.

	PL	FO	CC	CC-FO	*P*-value
Energy (kJ/day)	8449 ± 2882.1	8613 ± 1557.3	8625 ± 1755.7	8966 ± 1690.8	0.787
Carbohydrate (g/day)	227 ± 84.5	245 ± 58.6	241 ± 59.3	247 ± 69.4	0.380
Protein (g/day)	91.3 ± 35.8	86.4 ± 17.9	93 ± 21.6	101.8 ± 29.2	0.411
Starch (g/day)	159 ± 57.9	151 ± 49.6	133 ± 52.4	178.1 ± 57.6	0.141
Sugar(g/day)	43 (33, 108)	96 (56, 103)	80 (40, 106)	51 (32, 112)	0.241
Fibre (g/day)	24 (16, 32)	20 (15, 25)	20 (17, 23)	26 (20, 34)	0.453
Total fat (g/day)	71 (50, 93)	72 (55, 106)	83 (67, 91)	72 (55, 100)	0.673
Polyunsaturated fat(g/day)	11 (9, 17)	11 (6, 15)	12 (8, 17)	12 (8, 14)	0.383
Monounsaturated fat (g/day)	29 (19, 35)	28 (22, 39)	30 (25, 34)	29 (22, 41)	0.841

Data are reported as mean ± SD or median (25^th^ and 75^th^ percentile) as appropriate. Significant changes in dietary intake between the groups (ANOVA), P < 0.05. SD- Standard deviation.

### Postprandial glucose concentrations

**Figure 2:**
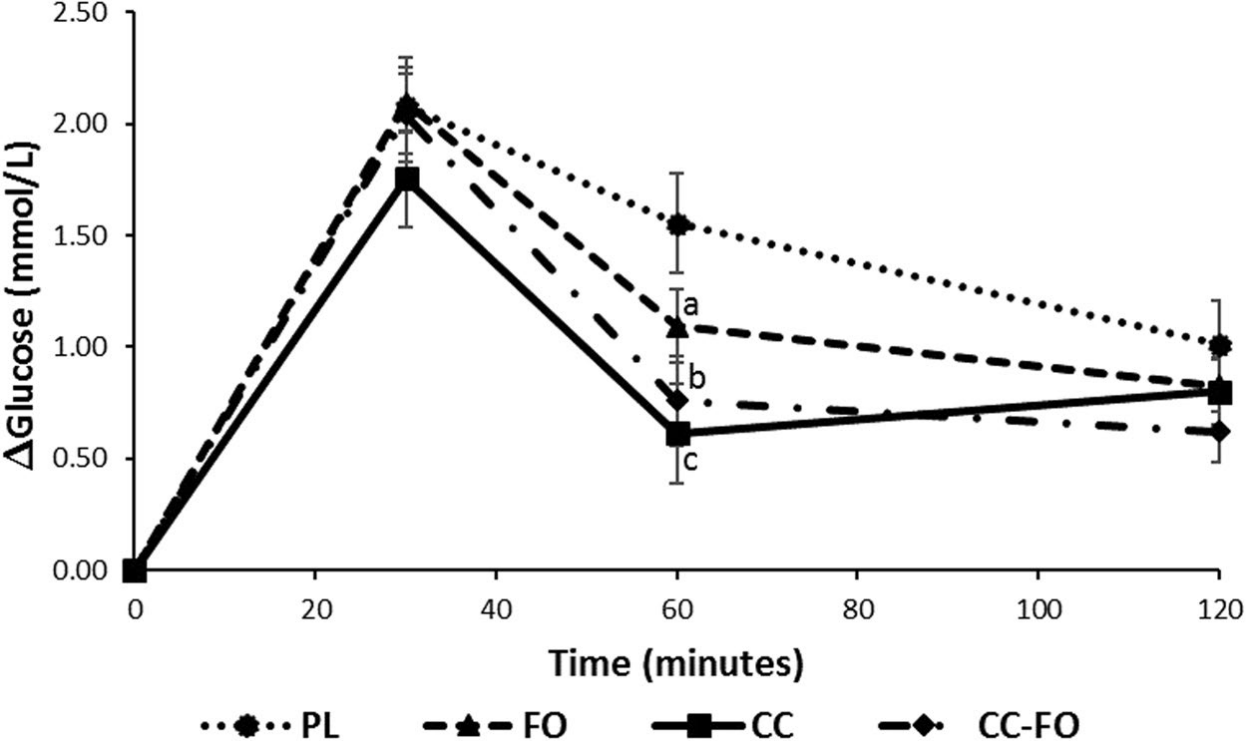
Changes in the mean blood glucose concentrations (∆glucose) over 120 minutes. Data are reported as Mean ± SEM. Lower case letters represent the significance difference between PL and active treatment groups ((**a**) P = 0.046; (**b**) P = 0.002; (**c**) P = 0.0007). PL, double placebo (n = 15); FO, fish oil (n = 15); CC, curcumin (n = 15); CC + FO, curcumin plus fish oil (n = 15).

There were no significant differences observed between the baseline glucose concentrations at the start of each of the 4 test days (Table 3). Increase in glucose concentrations post-meal consumption was lower in the curcumin treatment group at all the time points compared with the placebo group. There was no difference between the change in glucose concentrations between placebo and the fish oil groups. Two-way repeated measures analysis of variance (ANOVA) model with time and treatment was significant (P = 0.005) for change(Δ) in glucose concentrations (Fig. 2) and change area under the curve (AUC) for glucose (P = 0.007) (Table 3). Although there was 15.7% reduction in glucose concentrations with curcumin at 30 min compared with placebo, it did not reach significance (P = 0.208). Pair-wise analysis indicated 61%, 51% and 30% lower ∆glucose concentrations at 60 min in the curcumin (P = 0.0007), curcumin + fish oil (P = 0.002) and FO (P = 0.046) groups respectively, compared with placebo group. No significant differences were observed between curcumin, fish oil, curcumin + fishoil and placebo at 120 min. There were no significant differences observed between the curcumin, fish oil and curcumin + fish oil at all the three-time points, however the magnitude of the effect on ∆glucose reduction was different between the three treatments. Change AUC (0–120 min) for glucose was significantly lower with curcumin (36.2%; P = 0.003) and curcumin + fishoil (29.9%; P = 0.004), but not with fish oil (15.4%; P = 0.105) (Table 3). Change AUC for glucose were not significantly different between the active treatment groups.

**Table 3:** Baseline values and area under the curve (AUC) for change in the glucose, insulin and triglycerides in response to PL, FO, CC and CC-FO.

Parameters	Time (min)	PL	FO	CC	CC-FO	Model^a^ (time x treatment) P value	PL^b^ VS FO	PL^b^ VS CC	PL^b^ VS CC-FO
Fasting glucose (mmol/L)	0	5.3 ± 0.3	5.2 ± 0.37	5.4 ± 0.45	5.4 ± 0.48	0.441	—	—	—
Glucose change AUC (mmol. min/L)	0–120	2.7 ± 1.1	2.2 ± 1.2	1.73 ± 0.7	1.9 ± 1.0	0.007	0.105	0.003	0.004
Fasting insulin (mIU/L)	0	7.6 ± 2.5	8.2 ± 2.5	8.8 ± 3.7	9.0 ± 3.1	0.104	—	—	—
Insulin change AUC (mIU. min/L)	0–120	69.9 ± 53.1	57.4 ± 41.9	51.4 ± 45.1	51.9 ± 31.0	0.02	0.140	0.01	0.03
Fasting triglycerides (mmol/L)	0	1.0 ± 0.5	1.0 ± 0.3	1.1 ± 0.70	1.01 ± 0.38	0.534	—	—	—
Triglycerides change AUC (mmol. min/L)	0–120	0.3 (0.2, 0.5)	0.4 (0.2, 0.6)	0.2 (0.1, 0.6)	0.52 (0.33, 0.72)	0.100	—	—	—

Data are presented as mean ± SD or median (25^th^ and 75^th^ percentile). AUC – area under the curve. PL-double placebo, FO- fish oil alone, CC- curcumin alone, CC-FO- curcumin plus fish oil. ^a^Output from two-way repeated measures analysis of variance. ^b^Output from pair-wise analysis between the two groups.

### Postprandial insulin

**Figure 3:**
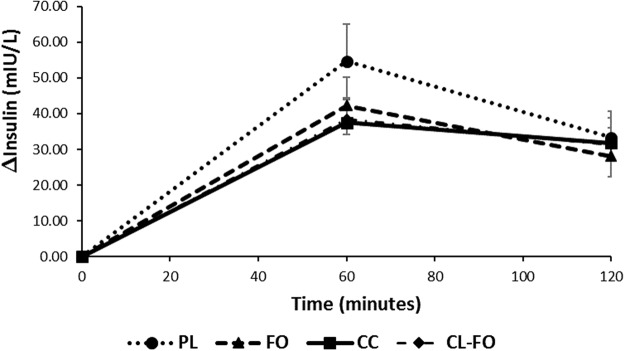
Changes in the mean serum insulin concentrations (∆insulin) over 120 minutes. Data are reported as Mean ± SEM. PL, double placebo (n = 15); FO, fish oil (n = 14); CC, curcumin (n = 15); CC-FO, curcumin plus fish oil (n = 15).

There were no significant differences observed between the baseline insulin concentrations at the start of each of the 4 test days (Table 3). Mean changes in insulin concentrations at all the time points were lower following curcumin treatment. However, two-way repeated measures ANOVA indicated that the model with time and treatment did not reach significance for the change in the insulin (∆insulin) concentrations (P = 0.168) (Fig. 3), but the change AUC for Insulin was significant (P = 0.03). Pairwise analysis shown mean change in AUC values for insulin was significantly lower only with curcumin (26.5%, P = 0.01) and curcumin + fish oil treatments (25.8%, P = 0.03) but not with fish oil treatment (18%, P = 0.140), when compared with placebo. There were no significant differences observed between the effects of curcumin, fish oil and curcumin + fish oil treatments on ∆insulin concentrations.

### Postprandial triglycerides

There were no significant differences observed between the baseline triglyceride concentrations at the start of each of the 4 test days (Table 3). As expected, the postprandial triglyceride concentrations were increased at all the time points following meal consumption compared with baseline. Two-way repeated measures ANOVA change with time and treatment model for change in triglyceride concentrations (∆triglycerides) did not reach significance (P = 0.074). Similar results were observed with for the change AUC triglycerides (P = 0.66).

### Confounding variables

No significant interaction was observed between gender and treatment effect for ∆glucose at 60 mins (P = 0.795), change AUC glucose (p = 0.995), change AUC insulin (p = 0.305) or ∆triglycerides at 60 min (0.958). No significant interaction between treatment effect and physical activity or BMI for ∆glucose at 60 mins (interaction with physical activity: p = 0.604; interaction with BMI: p = 0.701); change AUC glucose (interaction with physical activity: p = 0.173; interaction with BMI: p = 0.365),or change AUC Insulin (interaction with PA: p = 0.969; interaction with BMI: 0.06). Physical activity has a significant interaction with ∆triglycerides (0–120 min; p = 0.002) and change AUC (r = −0.33, p = 0.003) for triglycerides.

## Discussion

Results presented in the current study demonstrate that curcumin, but not LCn-3PUFA, has the potential to effectively reduce postprandial rise in glucose and insulin concentrations. Only curcumin and curcumin + fish oil significantly lowered the change AUC of the glucose and the amount of insulin (AUC) required to control postprandial rise in the blood glucose concentrations compared to the PL. Significant reduction in ∆glucose was observed with curcumin (61%) and curcumin + fish oil (50%) treatments.

Preclinical studies indicate that curcumin (various dose ranges, 60–300 mg/kg body weight) has the potential to regulate oxidative enzymes, suppress expression of pro-inflammatory cytokines, decrease hepatic glucose production and inhibit glycogenolysis by increasing the activation of AMPK, thereby, providing several putative mechanisms by which curcumin can control blood glucose levels^26^. Substantiating these reports, a recent meta-analysis^15^ with eleven randomised controlled trials, indicated that four or more weeks of supplementation with curcumin/and curcuminoids supplementation can effectively reduce the fasting glucose (−8.88, 95% CI: [−5.04 to −2.72] mg/dL, *p* = 0.005) and glycated haemoglobin levels (−0.54, 95% CI: [−1.09 to −0.002]%, *p* = 0.049). However, the exact mechanisms by which curcumin exhibits these clinical effects is still not clear. Curcumin supplemented through diets in rodent models has been shown to reduce the AUC for glucose in glucose tolerance test^5^.

Curcumin has been barely explored in the clinical trials to determine its effects on PBG control, however, as evident from the preclinical and *in vitro* studies, multiple mechanisms including stimulation of incretins (glucagon like peptide-1, GLP-1) and inhibition of digestive enzymes (α-glucosidase and α-amylase) for achieving control in post-meal glycaemia may exist. GLP-1 is known to influence gut motility, inhibit glucagon secretion and augment glucose-stimulated insulin secretion in the pancreas^27^. Curcumin has been previously shown to be a GLP-1 secretagogue in the pre-clinical studies^6,28^. In rodent models, curcumin improved glucose tolerance via stimulation of GLP‐1 secretion following intraperitoneal glucose injections^28^. Docking studies^29^ have shown the binding capacity of curcumin/curcuminoids to the carbohydrate digestive enzymes. We used a liposomal formulation of curcumin in the current study. Liposomes are susceptible to the physiological factors such as gastric acid, bile salts and lipases, which may disrupt the liposomal integrity and result in leakage of curcumin from the core^30^. Indeed, the magnitude of binding will depend on the degree of release and availability of free curcumin from liposomes in the gut lumen.

In contrast with our findings, a previous study involving turmeric extract (*Curcuma longa*)^31^ as the dietary source of curcumin, has shown to be ineffective in lowering PBG concentrations. However, given the fact that dose (15 capsules, each containing 400 mg turmeric extract), glucose load (75 g glucose), and the study participant characteristics (smokers and snuff users) were different to our study, it is not surprising that the results were controversial to current findings.

In animal models of insulin resistance, oral administration of curcumin has been shown to significantly improve the insulin sensitivity and glucose metabolism^5^. Curcumin has also been reported to exhibit reductions in fasting insulin and insulin resistance in individuals with pre-diabetes^11^. Reduced rise in meal-induced insulin by curcumin in the current study compared to the placebo group is suggestive of the beneficial effects of curcumin on insulin resistance possibly by regulation of the post-meal insulin response and/or preservation of the β-cell function.

In line with previous studies, physical activity was inversely correlated with plasma triglycerides^32^. ANCOVA analysis indicated that physical activity has significant interaction with treatment effects on triglycerides. The effects of curcumin on ∆ triglycerides in two-hour period did not reach significance. Given the fact that our hypothesis was primarily based on evaluation of curcumin and/or LCn-3PUFA on PBG, the two-hour period was insufficient to demonstrate effects on post-prandial triglycerides. A postprandial study design with time longer than 2-hours is necessary to study the effects of curcumin on postprandial lipids.

Replacement with LCn-3PUFA or meals rich in LCn-3PUFA has been shown to influence the insulin sensitivity without affecting the glucose metabolism in *in-vitro*^32,33^ and pre-clinical studies^34^. On the other hand, clinical trial reported no effect of LCn-3PUFA on insulin sensitivity or PBG^35,36^, leading to ambiguity and necessity for further research. Interestingly fish oil significantly reduced the ∆glucose at 60 min by 30% compared to the placebo. However, there was no effect of fish oil either on change AUC of the glucose or insulin or triglycerides. Pharmacokinetic studies indicate the plasma concentrations of LCn-3PUFA are increased significantly at 2 hours and 4 hours and peak at 6 hours post-supplementation^37^. The current study failed to demonstrate any effects of LCn-3PUFA in the limited two-hour study period. A longer study time may be necessary to evaluate the effects of LCn-3PUFA and any of its complementarity with curcumin on PBG and insulin response.

We demonstrated that curcumin has the potential to lower PBG and insulin responses, thus providing a new approach for glycaemic control with curcumin supplementation. These results can be further substantiated by elucidating mechanisms and pharmacokinetic studies on how exactly curcumin controls the post-meal glucose concentrations. Two-hour study duration was a limiting factor to study the benefits of combining curcumin with LCn-3PUFA on postprandial glycaemic parameters. Long term supplementation trials with curcumin and LCn-3PUFA are warranted to determine the combined effects of curcumin and LCn-3PUFA on post-prandial glycaemic control.

## Methods

### Subjects

Sixteen apparently healthy men and women were recruited from the Hunter Region, New South Wales, Australia via displaying recruitment flyers and media publicity. They were screened through telephone interviews based on the inclusion criteria: 18–45 years of age and BMI less than 30 kg/m^2^. Exclusion criteria included diagnosis with any chronic metabolic disease (type 2 diabetes and cardiovascular disease), auto-immune disease (rheumatoid arthritis, lupus, type 1 diabetes), liver disease, history of severe neurological diseases or seizures, currently on any medications/supplements known to influence blood glucose concentration, pregnancy or planning to become pregnant, breastfeeding, unwilling to provide informed consent and sensitivity/intolerance to curcumin, fish oil, and/or food products (dairy products, peanuts, wheat, protein and gluten). The study protocol was approved by the University of Newcastle Human Research Ethics Committee (H-2014-0385). All participants gave their written informed consent before entering the study. All the procedures were performed in accordance with relevant guidelines and in accordance with approved protocol by the University of Newcastle Human Research Ethics Committee. The study was registered on Australian New Zealand Clinical Trials Registry (ANZCTR) under study number ACTRN12618000047291 (date of registration: 16/01/2018).

### Standard meal

On the test days, participants were provided with standard breakfast: two slices of toasted white bread (62 g), peanut butter (22 g) and chocolate flavoured milk (250 mL, brand name; Oak). The macronutrient composition of this meal is 56 g carbohydrate, 22 g fat and 21 g protein. Participants consumed the test meal within 15 min.

### Study design and study products

This randomised, placebo controlled, single blinded (participants), cross over study consisted of 4 test days, each separated by a wash out period of one week. Following the screening, the eligible participants were asked not to change their dietary habits and concentration of physical exercise before the test days and during the washout periods. They received necessary instructions for each test day: 12-h overnight fasting, avoiding high caloric meal, alcohol consumption, and any vigorous physical activity 24-h before the test days. They were also asked to collect a 24-hour record of their previous day food intake. This dietary information was processed through FoodWorks Version: 8.0.3551 (Xyris Software (Australia) Pty Ltd) to check whether their dietary energy and macronutrients remained unchanged during the study period. Physical activity of the participants was assessed on the first test day using the International Physical Activity Questionnaire – short form designed to capture the frequency, duration and intensity of physical activity undertaken during the previous seven days. Participants were categorised as low physical activity level if they do not meet moderate (3 or more days of vigorous intensity activity of at least 20 minutes per day or 5 or more days of moderate intensity activity and/or walking of at least 30 minutes per day or 5 or more days of any combination of walking, moderate intensity or vigorous intensity activities achieving a minimum total physical activity of at least 600 metabolic equivalents per minute/week) or high(vigorous-intensity activity on at least 3 days achieving a minimum total physical activity of at least 1500 metabolic equivalents per minute/week or 7 days of any combination of walking, moderate intensity or vigorous intensity activities achieving a minimum total physical activity of at least 3000 metabolic equivalents per minute/week) criteria. Participants received a single dose of one of the following dietary supplements in presence of study investigators just prior to consuming the standardised breakfast, in a random order according to the computer generated sequence (block size of four): Placebo (PL, 2 placebo tablets matching for curcumin +2 placebo capsules matching for fish oil); curcumin (CC, 2 curcumin tablets +2 placebo capsules matching for fish oil); fish oil (FO, 2 fish oil capsules +2 placebo tablets matching for curcumin); and curcumin + fish oil (2 curcumin tablets +2 fish oil capsules). Curcumin used in the current study is commercially available MERIVA^®^ formulation that contains more than 20% curcuminoids (curcumin: demethoxycurcumin: bisdemethoxycurcumin in the ratio of 75:15:10) complexed with soy lecithin in a 1:2 weight ratio, delivering 90 mg in each tablet. Fish oil capsules (EPAX 1050TG) allocated to the study participants are high in DHA, each capsule delivering 120 mg EPA + 430 mg DHA.

### Test day protocol

After the participants arrival at the Nutraceutical Research Program clinical trial facility on the first test day in fasting state, medical history and demographic related information were obtained from participants via self-administered questionnaires. Body mass index (BMI) and body composition were determined using direct segmental multi-frequency bioelectrical impedance (InBody 230, Biospace Co., Ltd. Seoul, Korea). Blood samples were collected by finger prick for the measurement of fasting glucose concentrations (BG star glucometer, Sanofi). An 8-ml venous blood sample was also collected and sent to the accredited Hunter Area Pathology Service for the measurements of fasting serum insulin and triglycerides. No other food or drink was allowed during the 2 h period on the test days. Capillary and venous blood sampling was repeated at 30, 60 & 120 min and 60 & 120 min respectively.

### Statistics

The primary outcome for this study was ∆glucose concentrations at 60 min. Data from our previous study^38^ indicate that the difference in the response of subjects is normally distributed with standard deviation 0.11. With the expected 10% difference in the mean ∆glucose response at 60 min with a probability (power) 0.8, a sample size of n = 12 subjects were required in a cross-over design. The Type I error probability associated with this test of this null hypothesis is 0.05. The data of all variables included in the analysis were tested for normality by deriving studentized residuals, kernel density plots and Shapiro-Wilk’s tests and are presented as mean ± SD or median (interquartile range) as appropriate. Log transformation was used for variables with non-normal distribution. Differences between fasting concentrations of the biochemical variables, diet and macronutrient intake were evaluated by repeated measures analysis of variance. Change AUC’s (0–120) for postprandial glucose, insulin and triglyceride responses were constructed using trapezoid rule. Differences between the study interventions were tested for statistical significance using two-way repeated measures ANOVA. When this analysis was significant, a paired t-test or Wilcoxon signed-rank test was performed to compare the interventions pairwise. Analysis of covariance (ANCOVA) and two-way ANOVA was used to evaluate the effects of confounding variables on primary outcomes. Significance was set at p < 0.05. All statistical analyses were conducted using Stata version 14.1 (StataCorp, Texas, USA).

## Electronic supplementary material

Clinical Protocol

CONSORT Checklist
