# Supplementary material for: Curcumin alleviates postprandial glycaemic response in healthy subjects: A cross-over, randomized controlled study
Source: Sci Rep. 2018 Sep 12;8:13679. doi: 10.1038/s41598-018-32032-x (PMC6135827; doi:10.1038/s41598-018-32032-x)
Supplement: Supplementary file 1 — Clinical Protocol [file 41598_2018_32032_MOESM1_ESM.docx]

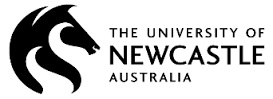


**Clinical Research Protocol**

**NUTRACEUTICALS RESEARCH GROUP**

***NUTRITIONAL SUPPLEMENTS FOR PREVENTION OF TYPE II DIABETES***

| **Investigating Institution:** | *University of Newcastle*  *Faculty of Health & Medicine*  *School of Biomedical Sciences & Pharmacy*  *Nutraceuticals Research Group*  *Callaghan, NSW 2308*  *Australia* |
| --- | --- |
| **Sponsor 1:** | *XXXXX* |
| **Sponsor 2:** | *XXXXX* |
| **Study Products:** | *Meriva – Indena (Curcumin), EPAX 1050 TG/N (Omega 3 fatty acids)* |
| **Protocol Number:** | *1.5* |
| **Ethics Reference:** | *TBC* |

**Initial version: 07 November 2014**

**Amended: 08 April 2015**

**Amended: 04 May 2015**

**Amended: 22 June 2015**

**Amended: 23 AUG 2017**

**Amended: 07 SEP 2017**

Investigators

| Principal Investigator | **Prof Manohar Garg, PhD, MND, MSc, BSc (Hons), RNutr** |
| --- | --- |
| Company | University of Newcastle |
| Address | Nutraceuticals Research Group  School of Biomedical Sciences & Pharmacy  University of Newcastle  Callaghan NSW 2308  Australia |
| Phone | 61-2-4921 5647 |
| E-mail | manohar.garg@newcastle.edu.au |

| Co-Investigator | **Dr Sham Acharya, FRCP Edin, FRACP** |
| --- | --- |
| Company | Health, Hunter New England Local Health District |
| Address | Locked Bag 1, Hunter region mail centre  NSW 2310 |
| Phone | 61-2-4922 3366 |
| Email | Shamasunder.Acharya@hnehealth.nsw.gov.au |

| Co-Investigator | **Mr Rohith Thota, BPharm, MPharm, PhD Candidate** |
| --- | --- |
| Company | University of Newcastle |
| Address | Nutraceuticals Research Group |
| Phone | School of Biomedical Sciences & Pharmacy  University of Newcastle  Callaghan NSW 2308  Australia  61-2-4921 5638 |
| Email | RohithNagendra.Thota@uon.edu.au |

Manufacturer and Packaging and Labeling

| Manufacturer |  |
| --- | --- |
| Company | **Indena S.p.A** |
| Address | Viale Ortles 12  20139 Milan, Italy |
| Phone | +3902574961 |
| Fax | +390257404620 |
| Company  Address  Telephone  Telefax  Suppliers  Telephone  Telefax | **EPAX Norway AS**  Po box 7047  No- 6028 Alesund  Norway  +4770135960  +4770135961  **Pathway international Pty.**  14 Rodborough Rd, Level 2  Frenchs Forest NSW 2086  (02) 9984 2700  +61 2 9984 2755 |
|  |  |

Laboratories

| **For Curcumin, n-3PUFA and other biomarkers** | |
| --- | --- |
| Company | University of Newcastle |
| Department | Nutraceuticals Research Group |
| Address | School of Biomedical Sciences & Pharmacy  University of Newcastle  Callaghan, NSW 2308  Australia |
| Phone | +61 2 4921 5636 |
| E-mail | Melissa.fry@newcastle.edu.au |
| **For blood lipids, glucose, insulin, HbA1c** |  |
| Company | **Hunter New England Area Pathology Services (HNEPS)** |
| Department | **Pathology North** |
| Address | Pathology North  Locked Bag 1  Hunter Region Mail Centre, NSW 2310  Australia |
| Phone | +61 2 4921 4000 |
| Fax | +61 2 4921 4440 |

Synopsis

| Title | | Nutritional supplements for prevention of type 2 diabetes | | |
| --- | --- | --- | --- | --- |
| Protocol Number | | 1.0 | Study Acronym | N/A |
| Version | | 1.1 | Date | 7 NOV 2014 |
| Sponsors | | University of Newcastle | | |
| Study Phase | | II |  |  |
| Application field | | Diabetes | | |
| Primary objective | | A randomized controlled trial will be undertaken with primary aim to evaluate the potential of curcumin and / or omega-3 polyunsaturated fatty acid (n-3PUFA) for improving glycemic profiles (glucose, insulin, HOMA-IR, HbA1c) and insulin sensitivity in individuals with pre-diabetes. | | |
| Secondary objectives | | To assess effect of curcumin and / or n-3PUFA on  - lipid profile  - CRP and IL-6  - Adiponectin and leptin  - Erythrocyte fatty acid composition  -To determine if there is any improvement in bioavailability of Curcumin when co-administered with n-3PUFA | | |
| Study Design | | 2x2 factorial, randomized, double blind, placebo controlled study | | |
| Planned Sample Size | | N=20 each group, total number of participants = 80 | | |
| Number of Sites | | 1 | | |
| Investigational Products | | Curcumin , n-3PUFA | | |
| Dosage and Regime | 1) Placebo: 4 capsules/day (2 each for curcumin and fish oil placebos)  2) Curcumin (2 cap @500 mg each) providing 180 mg curcumin plus 2 placebo capsules/day  3) n-3PUFA (2 cap @1000mg each) providing 1.2g EPA/DHA plus 2 placebo capsules/day  4) Curcumin (2 cap @500 mg each providing a total of 180 mg curcumin) and n-3PUFA (2 cap @1000mg each providing a total of 1.2g EPA/DHA) per day | | | |
| Duration of intervention | | 12 weeks | | |
| Efficacy Parameters | | ***Primary:***   - HbA1c - Fasting plasma glucose and insulin levels to determine the HOMA-IR   ***Secondary:***   - Fasting total, HDL and LDL cholesterol and triglycerides - Anthropometric measures (BMI and Weight, waist-hip ratio, waist circumference) - IL-6 , Fatty acids - Adiponectin and leptin | | |
| Safety Parameter | | Adverse Event reporting | | |
| Study Procedures | | Screening assessment (visit 1)   - Volunteers will be recruited through public advertisement and an existing database of volunteers that have been identified as likely to meet the inclusion criteria. - They will attend Nutraceutical Research Group having fasted for at least 10hr. - Volunteers who meet the study inclusion criteria will be enrolled in the study. - A blood sample for blood glucose, insulin, HbA1c and other biomarker for the baseline levels will be obtained.   Follow Up visit (visits 2)   - Follow up visit include adverse events monitoring, anthropometrical measurements, diet and physical activity   Post intervention visit (Visit 3)   - They will attend the Nutraceutical Research Group having fasted for at least 10hr - A blood sample for post intervention measurements will be obtained. | | |
| Subject Selection Criteria | | Inclusion criteria:   - Age – 30-70; gender – both males and females - No participation in any clinical trial for at least 3 months - An HbA1c of 5.7% - 6.4% - Impaired Glucose Tolerance (IGT):   *ƒ-*2-hour OGTT plasma glucose ≥7.8 mmol/ L and <11.1 mmol/L   - Impaired fasting glucose (IFG):   Fasting plasma venous glucose measurement 6.1–6.9 mmol/L   - 12 or more score or High risk individuals in AUSDRISK assessment tool - BMI 25 – 45   Exclusion criteria:  Volunteers will be excluded if they have/are   - Pregnancy or lactation - Established type 2 diabetes - Allergic to sea foods - People with gall bladder problems - People with pace maker implants - Currently on medication with Aspirin and Warfarin - History of severe neurological diseases or seizures - History of new investigational drug three months prior to this trial - Consuming more than 2 serve of oily fish per week - Taking regular dietary supplements known to influence blood glucose level - Unwilling to fast for 10hr before obtaining blood sample | | |
| Statistical Considerations | | Sample size determination  Seventeen participants in a 2x2 factorial study design will give 80% power to detect a 0.5 units drop in HbA1c at alpha = 0.05. To allow for dropouts we will recruit 4x20 = 80 participants according to the inclusion criteria. Baseline data Baseline measures will be used as covariates. Gender, BMI and other potentially confounding variables such as weight and duration of pre-diabetes may be added as covariates if they are significantly correlated with the outcome measures. Treatment effects All the data relating the significant effects of Curcumin and / or n-3PUFA are expressed as mean ± SEM. The effect of interventions on the blood glucose levels and HbA1C between groups will be estimated by using two-way ANOVA with post hoc comparisons (Tukey’s honestly significant difference). Significance (*P*-value set at 0.05) indicates the changes from the baseline values. Changes from the baselines will be determined using nonparametric analysis (Wilcoxon’s signed ranked test). This statistical analysis helps in determining whether there will be a significant main effect for each independent variable by testing for between subject effects. The statistical analysis by this method will be performed to evaluate the synergistic effects between Curcumin and n-3PUFA. | | |

**Table of Contents**

1 Introduction 1

1.1 Background 1

1.2 Investigational Product 1

1.3 Dose Rationale and Risk/Benefits 2

2 Study Objectives and endpoints 2

2.1 Primary objective 2

2.2 Primary endpoints 2

2.3 Secondary endpoints 2

3 Sites and Subjects 2

3.1 Study site 2

3.2 Number of subjects 2

3.3 Inclusion criteria 2

3.4 Exclusion criteria 3

4 Study Design and Subject Assignment 3

4.1 General Design 3

4.1.1 Duration of Study 3

4.2 Randomisation 3

4.3 Unblinding procedure 3

5 Subject Selection and Withdrawal 4

5.1 Subject Recruitment and Screening 4

5.2 Early Withdrawal of Subjects 4

5.2.1 When and How to Withdraw Subjects 4

5.2.2 Data Collection and Follow-up for Withdrawn Subjects 4

6 Investigational Product 5

6.1 Description 5

6.2 Treatment Regimen 5

6.3 Preparation and Administration of Investigational Product 5

6.4 Prior and Concomitant Therapy 5

6.5 Packaging 5

6.6 Blinding of study treatment 5

6.7 Receiving, Storage, Dispensing and Return 5

6.7.1 Receipt of Investigational Product 5

6.7.2 Storage 6

6.7.3 Dispensing of Investigational Product 6

6.7.4 Return or Destruction of Investigational Product 6

7 Study Procedures 6

7.1 Schedule of assessments 6

7.2 Screening and eligibility 7

7.2.1 Obtaining informed consent 7

7.2.2 Assessment of eligibility at screening/baseline visit 1 7

7.3 Intervention phase 9

7.3.1 Visits 2 & 3 9

7.4 Outcome assessments 9

8 Sample Collection and Assessments 9

8.1 Blood sample 9

8.1.1 Blood sample collection 9

8.1.2 Blood sample assessment 9

9 Safety Evaluation 10

9.1 Potential risks 10

10 Statistical Plan 10

10.1 Sample Size Determination 10

10.2 Statistical Methods 10

10.2.1Baseline data 10

10.2.2Treatment effects 10

10.3 Subject Population(s) for Analysis 11

11 Safety and Adverse Events 11

11.1 Definitions 11

12 Data Handling and Record Keeping 12

12.1 Confidentiality 12

12.2 Records Retention 13

13 Study Monitoring, Auditing, and Inspecting 13

13.1 Study Monitoring Plan 13

13.2 Auditing and Inspecting 13

14 Ethical Considerations 13

15 Investigator Responsibilities 14

15.1 Ethics committee approval 14

15.2 Subject information and obtaining consent 14

15.3 Withdrawals of subjects 14

15.4 Subject confidentiality 15

15.5 Training of staff 15

15.6 Protocol amendments 15

15.7 Case Report Form 15

15.8 Source document and source data verification 15

16 Study Finances 15

16.1 Subject Stipends or Payments 15

17 Study Report and Publication Plan 15

18 Archiving 16

19 References 16

# Introduction

This document is a protocol for a human research study. This study is to be conducted according to NHMRC and international standards of Good Clinical Practice (International Conference on Harmonization guidelines), and Institutional research policies and procedures.

## Background

The prevalence of type 2 diabetes and its associated complications represents major global health hazard . According to the Diabetes Australia, approximately 280 individuals develop diabetes daily and the number of diabetics reached 1.1 million with 100,000 new cases diagnosed per annum (Diabetes Australia website, 6/11/2014). The fundamental problem in preventing the type 2 diabetes is the restoration of insulin sensitivity in tissues and improving the glycemic index, which is already deregulated in the pre-diabetic individuals.

Pre-diabetes is a condition in which blood glucose level are higher than normal, but not high enough to be diagnosed as type 2 diabetes. Pre-diabetes includes individuals with impaired fasting glucose or impaired glucose tolerance and elevated HbA1c levels. Insulin resistance and β-cell dysfunction are characteristic pathological conditions of pre-diabetes ([1](#_ENREF_1), [2](#_ENREF_2)). Impaired fasting glucose reflects hepatic insulin resistance and the impaired glucose tolerance reflects the high muscle resistance. Impaired glucose tolerance shows reduction in both late and early phase insulin secretion, whereas impaired fasting glucose reflects reduced early phase insulin secretion([3](#_ENREF_3)).

***Role of inflammation and insulin resistance in diabetes***

Inflammation has been demonstrated as the common and initial pathophysiological mechanism in the development of diabetes via dysregulation of adipokine levels, glucotoxicity, lipotoxicity leading to systemic insulin resistance in individuals ([4](#_ENREF_4)). Targeting inflammation in individuals who are at a risk of type 2 diabetes may lead to improvement in glycemic profile and insulin sensitivity ([5](#_ENREF_5)). In the present study we propose to evaluate the complimentary and/or synergistic effects of curcumin and n-3PUFA in modulating inflammatory biomarkers for improving the insulin resistance and glycemic profile in individuals with pre-diabetes for delaying the progression to type 2 diabetes.

## Investigational Product

Curcumin and n-3PUFA

***Pre-clinical and Clinical Data***

A key mechanism by which curcumin may exhibit its salutary effect is by modulating the inflammatory pathways involved in pathogenesis of type 2 diabetes ([6](#_ENREF_6)). Curcumin down regulates the expression of TNFα and NFκB which are precursors for expression of a wide range of inflammatory mediators ([7](#_ENREF_7)). Curcumin is well tolerated in the individuals and proven to be safer even at higher doses in human intervention trials ([8](#_ENREF_8)). Poor bioavailability of curcumin in humans is a barrier in properly evaluating its health benefits.

There is increasing evidence from literature that certain bioactive nutrients such as omega-3 fatty acids from fish and fish oil found to be beneficial in improving the glucose tolerance and insulin sensitivity in preclinical studies by modulating the inflammatory pathways, increasing the mitochiondrial biogenesis in adipocytes, decreasing glucose production and improving the insulin sensitivity ([9-11](#_ENREF_9)) and to a limited extent in clinical studies ([12-14](#_ENREF_12)). In this present study we evaluate the synergistic or complimentary effect of curcumin and n-3PUFA on glycemic profile and insulin resistance.

***Rationale for combination of Curcumin and n-3PUFA:***

Curcumin bioavailability can be improved when co-administered with lipids ([15](#_ENREF_15)) by improving its gastrointestinal absorption. Co-administration of fish oil containing n-3PUFA with curcumin has the potential to not only enhance curcumin bioavailability but also to potentiate its biological effects.

## Dose Rationale and Risk/Benefits

## Low dose combination therapy is believed to be relatively safe and beneficial than high dose single drug with toxic effects. Doses of curcumin and omega 3 fatty acids taken under investigations was well tolerated and reported no adverse events in the previous studies ([16](#_ENREF_16), [17](#_ENREF_17)).

# Study Objectives and endpoints

## Primary objective

A clinical evaluation of a curcumin supplemented with or without omega-3 polyunsaturated fatty acids (n-3PUFA) for prevention of type 2 diabetes

## Primary endpoints

- Improvement in blood levels of HbA1c

## Secondary endpoints

- Fasting blood glucose, insulin and HOMA-IR score
- Fasting total, HDL and LDL cholesterol and triglycerides
- Anthropometric measures (BMI and Weight, waist-hip ratio, waist circumference)
- IL-6 , Fatty acids
- Adiponectin and leptin
- Bioavailability of curcumin when co-administered with n-3PUFA

# Sites and Subjects

## Study site

Nutraceuticals Research Group, Level 3 Medical Science Building, University of Newcastle, Callaghan, New South Wales, Australia

## Number of subjects

20 participants in each group – total n=80 people with pre-diabetes

## Inclusion criteria

• Age – 30-70; gender – both males and females

• No participate in any clinical trial at least 3 months

• An HbA1C of 5.7% - 6.4%

• Impaired Glucose Tolerance (IGT):

- ƒ-2-hour OGTT plasma glucose ≥7.8 mmol/ L and <11.1 mmol/L

• Impaired fasting glucose (IFG):

- Fasting plasma venous glucose measurement 6.1–6.9 mmol/L

- 12 or more score or High risk individuals in AUSDRISK assessment tool
- BMI 25 – 45

## 3.4 Exclusion criteria

Volunteers will be excluded if they have

• Pregnancy or lactation

• Established type 2 diabetes

• Allergic to sea foods

• People with gall bladder problems

• People with pace maker implants

• Currently on medication with Aspirin and Warfarin

• History of severe neurological diseases or seizures

• History of new investigational drug three months prior to this trial

• Consuming more than 2 serve of oily fish per week

• Taking regular dietary supplements known to influence blood glucose level

• Unwilling to fast for 10hr before obtaining blood sample

# Study Design and Subject Assignment

## General Design

A 2x2 factorial, randomized, double blind, placebo controlled study of 12 weeks duration will be conducted in accordance with ICH GCP standards. The trial will be approved by the University of Newcastle Human Research Ethics Committee and registered with Australian New Zealand Clinical Trials Registry (ANZCTR).

The study will be conducted according to International Conference on Harmonization guidelines for Good Clinical Practice and University of Newcastle research policies and procedures.

### Duration of Study

Twelve weeks

## Randomization

Allocation to treatments will be based on the computer generated block randomization method to ensure well-balanced groups.

|  | **Curcumin Group** | **Placebo Group** | **Total** |
| --- | --- | --- | --- |
| **N-3PUFA Group** | 20 | 20 | 40 |
| **Placebo Group** | 20 | 20 | 40 |
| **Total** | 40 | 40 | 80 |

## Unblinding procedure

Should the need for unblinding in the curcumin or n-3PUFA group vs. placebo group arise, subjects may inform the study coordinator or anyone from the research team. The study coordinator will inform the principal investigator of all AEs and SAEs, who will then follow procedures for unblinding as necessary and notify the relevant bodies. Depending on the nature of AE or SAE, it may be necessary for treatment to cease and/or for the participant to be withdrawn from the study.

For emergency situations, subjects will be given an emergency contact card, which will provide general study information and contact information for the unbiased randomizers.

# Subject Selection and Withdrawal

## Subject Recruitment and Screening

Recruitment for this project will commence following approval of the protocol by the Human Ethics Committee of University of Newcastle. It is anticipated that the subject activity will last 3 months. Participants will be recruited from the Hunter Region in NSW using media advertising and social media approved by the University of Newcastle Media and Marketing Department. Recruitment flyers will also be placed on noticeboards at the University of Newcastle, local pharmacies, local pathology centers (with permission). Participants will also be recruited from the Hunter Medical Research Institute (HMRI) Volunteer Register and as well as from clinics across John Hunter/Newcastle Community Health Centre and Belmont Hospital diabetes clinics. We will also recruit participants from Medicare local and GP practices database with consent from participating GPs.

Interested participants will contact the study coordinator who will send a Participant Information Statement and the Health, Diet & Lifestyle Questionnaire. Interested parties will return the completed Questionnaire along with a signed consent from the volunteer to allow the study investigators to use demographic and lifestyle details to help determine their suitability before visiting the Nutraceuticals Research Group for a screening/baseline visit (i.e. whether participant meets the specific study criteria).

## Early Withdrawal of Subjects

### When and How to Withdraw Subjects

Participants have the right to withdraw from the study at any time for any reason, without being obliged to give reasons and without penalty or loss of benefits they are entitled to. The investigator also has the right to withdraw participants from the study if it is in their best interest. Participants who withdraw prematurely from the study will not be replaced. However, data collected may be used in the data analysis if the participant has not withdrawn consent for collected data to be used.

Participants will be discontinued from the study if:

- The participant experiences a Serious Adverse Event (SAE) that is directly due to the active treatment.
- The participant or the participant’s attending physician requests that the participant be withdrawn from the study.
- The participant does not meet the enrolment criteria
- The participant is unwilling to comply to study protocol

### Data Collection and Follow-up for Withdrawn Subjects

Participant will reserve their right to withdraw the data that has been collected from them from being included in the data analysis. However, the collected data from withdrawn subjects cannot be destroyed and will be retained for the minimum storage period of 20 years as stated by National Health and Medical Research Council of Australia Research Management Policy.

No further data will be collected once they withdraw their informed consent.

# Investigational Product

## Description

Refer to Investigator Brochure.

## Treatment Regimen

The doses selected for this proposed study are similar to those used in previously published studies in adults shown to be safe and tolerable. Placebo and active capsules will be identical in shape and colour. Enrolled participants will be required to consume 4 capsules per day (two capsules in the morning and two capsules in the evening) as follows:

## 1) Placebo: 4 capsules/day (2 each for curcumin and fish oil placebos)

## 2) Curcumin (2 cap @500 mg each) providing 180 mg curcumin plus 2 placebo capsules/day

## 3) n-3PUFA (2 cap @1000mg each) providing 1.2g EPA/DHA plus 2 placebo capsules/day

## 4) Curcumin (2 cap @500 mg each providing a total of 180 mg curcumin) and n-3PUFA (2 cap @1000mg each providing a total of 1.2g EPA/DHA) per day

## Preparation and Administration of Investigational Product

Capsules will be taken orally with water along with the morning and evening meals.

The Investigator’ staff will instruct the intake of the capsules during participant’s visit to NRG.

## Prior and Concomitant Therapy

Regular medication and supplement intake will be recorded on the case report form at the start of the intervention. Any rescue therapies such as antibiotic or paracetamol consumed during the trial are permitted and must be documented in the case report form, including generic name, indication, total daily dose, route and time/duration of administration.

Doses and type of medication for pre-existing medication must remain constant during the trial.

Habitual diet and physical activity should be maintained throughout the study.

## Packaging

Capsules will be packaged by a research assistant not involved in data collection and labeled as supplement A-D and will be provided to the volunteers in random order. The doses that these letters correspond to will not be revealed to the investigators until after the data has been analyzed.

The code will be held by a member of the research clinic staff who is not involved in the study.

## Blinding of study treatment

Active and placebo treatment will be encapsulated and matched for color.

## Receiving, Storage, Dispensing and Return

### Receipt of Investigational Product

Upon receipt of the of the study treatment supplies, an inventory will be performed to verify that the shipment contains all the items noted in the shipment inventory before the receipt log filled out and signed by the person accepting the shipment. Any damaged or unusable study treatment supply in a given shipment (active capsules or placebo) will be documented in the study files. The investigator will notify the study sponsor of any damaged or unusable study treatments that were supplied to the investigator’s site.

### Storage

Investigational product will be carefully stored (under the responsibility of the investigator) in a locked, limited access area, safe and separated from drugs. Only authorized persons will have access to the storage place.

The capsules will be stored in sealed HD-PE bottles, stored at room temperature (15-25°C) in a dry place, protected from light and away from heat sources.

### Dispensing of Investigational Product

A Supplement Accountability Log will be used to document the dispensing of the dietary supplement or placebo to the subject. The Supplement Accountability Log will document the subject ID, initials, the amount of dietary supplement and date dispensed to the subject.

### Return or Destruction of Investigational Product

At the completion of the study, there will be a final reconciliation of study treatment supplies shipped, consumed, and quantity remaining. This reconciliation will be logged on the investigational product reconciliation form, signed and dated. Any discrepancies noted will be investigated, resolved, and documented prior to destruction of unused study treatment supplies. Investigational product destroyed on site will be documented in the study files.

# Study Procedures

## Schedule of assessments

| **Assessments** | **Visit 1** | **Visit 2** | **Visit 3** |  |  |  |
| --- | --- | --- | --- | --- | --- | --- |
|  | **Week 1** | **Week 6** | **Week 12** |  |  |  |
|  | **Screening** | **test** | **Test** |  |  |  |
| Informed consent | x |  |  |  |  |  |
| Randomization | X |  |  |  |  |  |
| Demographics | x |  |  |  |  |  |
| **Anthropometry** | x | X | X |  |  |  |
| **Blood sample** | x |  | x |  |  |  |
| **Food diary** | x | x | x |  |  |  |
| **Physical activity** | x | x | x |  |  |  |
| **Laboratory** |  |  |  |  |  |  |
| Blood glucose | x |  | X |  |  |  |
| Blood insulin | x |  | X |  |  |  |
| HbA1C | x |  | X |  |  |  |
| Plasma curcumin concentration |  |  | x |  |  |  |
| **Adverse Event reporting** | x | x | x |  |  |  |
|  |  |  |  |  |  |  |

## Screening and eligibility

### Obtaining informed consent

According to GCP-guidelines and also the Declaration of Helsinki, written informed consent must be obtained from subjects prior to participation in the trial. Subjects will voluntarily confirm their willingness to participate in the trial, after having been informed in writing and verbally of all aspects of the trial that are relevant to the subject's decision to participate. Subjects will be informed about requirements concerning data protection and have to agree to the direct access to their individual data. Subjects will sign an informed consent form for study participation. Before informed consent is obtained, the subject has to be provided sufficient time and opportunity to inquire about details of the trial and to decide whether or not to participate in the trial. All questions about the trial will be answered to the satisfaction of the subject. The original signed informed consent form will be kept with the study documentation. A copy of the signed informed consent document and participant information sheet must be given to the subject.

Participants that meet the study inclusion criteria will be required to attend the NRG 3 times throughout the study period (0^th^ week, 6^th^ week and 12^th^ week).

### Assessment of eligibility at screening/baseline visit 1

Schedule of baseline assessments is shown in Figure 7.2.2. Volunteers will arrive at the Nutraceutical Research Group following a 10hr fast (no food/beverage, except water) for further screening to determine study eligibility. Anthropometric measurements of height, weight and waist circumference will be obtained before obtaining blood sample.

**FIGURE 7.2.2** – Schedule of assessment for screening/baseline visit 1. BMI eligibility will be determined by height and weight within the range for study inclusion. Those who meet the study inclusion criteria will proceed for blood sampling. A fasting blood sample of blood glucose, insulin and HbA1c levels will be obtained after the screening visit.

## Intervention phase

### Visits 2 & 3

Schedule of assessment is shown in Figure 7.2.2 Participants will arrive at the Nutraceuticals Research Group at University of Newcastle for a follow up visit after 6 weeks. They will be enquired about adverse events, food and physical activity. Their capsule intake will be recorded will be reviewed and provided for next 6 weeks. The participants will revisit after 6 weeks at the end of the trial for post intervention blood sampling.

## Outcome assessments

• Fasting plasma glucose and insulin levels to determine the HOMA-IR

• HbA1c

• Fasting total, HDL and LDL cholesterol and triglycerides

• Anthropometric measures (BMI, waist circumference, waist-hip ratio, fat and lean body mass)

• CRP and IL-6

• Adiponectin and leptin

• Erythrocyte fatty acid composition

## Acute study

***PRIMARY*** ***OBJECTIVE:*** *A cross-over placebo controlled trial will be undertaken with primary aim to evaluate the effects of curcumin and/or omega-3 polyunsaturated fatty acid (n-3PUFA) on post prandial blood glucose levels in healthy individuals.*

***SECONDARY OBJECTIVES:***

*To assess effect of curcumin and / or n-3PUFA on post-prandial levels of*

- *Triglycerides*
- *Insulin*
- *leptin*
- *Glucagon*
- *Glucagon-like peptide 1 (GLP-1)*
- *Erythrocyte fatty acids*

***STUDY DESIGN****:*

*Cross over, placebo controlled study*

***PLANNED SAMPLE SIZE*** *N=15*

*With a standard deviation of 0.11, twelve participants in a cross over placebo controlled study will give 80% power to detect a 10% drop in Peak postprandial blood glucose levels at alpha = 0.05. To allow for 25% dropouts we will recruit additional 3 participants according to the inclusion criteria.*

***DURATION OF TRIAL****: 4 two and half an hour visits over 4 weeks.*

***STUDY PROCEDURES****:*

*Following the screening procedures, if potential participants agree to participate in this study, they will be asked to sign the participant consent form. Throughout the study, they will need to visit the Nutraceuticals Research Group on 4 occasions for two and half hours (one visit per week over 4 weeks):*

*At the initial (week 1) visit we will be asking them to do the following;*

*• Complete a brief medical questionnaire (provides information about your medical history, supplement intake and medications)*

*• Complete a physical activity questionnaire (provides information about your physical activity levels)*

*• Complete a 24-hour food recall (provides information about your dietary and nutrient intake)*

*• The day before your appointments you will be asked to refrain from physical activity and alcohol consumption and visit the trial site after an overnight fasting state of at the least for 10 hours.*

*• Consume the provided standard meal in the night before the visit day.*

*• Provide us with finger prick blood sample for testing glucose levels at 4 intervals (0, 30 min, 60 min, 120 min)*

*• Provide 8 mL of blood (collected through venous blood collection that involves three separate site punctures at each visit ) for 3 times in two hours (0, 1 hour, 2 hour)*

*Along With standard meal comprising 49% carbohydrate, 35% fat, 15% protein (250 mL Oak chocolate drink + 2 white bread slices (toasted) + 1 pat butter), each participant will be receiving the following :*

*1) 2 x placebo tablets (matching for curcumin) + 2 x placebo capsules (matching for fish oil)*

*2) 2 x 500 mg curcumin tablets + 2 x placebo capsules (matching for fish oil)*

*3) 2 x 1000 mg fish oil capsules + 2 x placebo tablets (matching for curcumin)*

*4) 2 x 500 mg curcumin tablets + 2 x 1000 mg fish oil capsules*

*Placebos are tablets or capsules that look like the tablets or capsules under investigation, but contain no active ingredients.*

*At the remaining visits (week 2, 3 & 4) we will be asking them to do the following:*

*• Complete a physical activity questionnaire*

*• Complete a 24-hour food recall*

*• Provide Finger prick and venous blood sample (similar procedures to the first visit) after an overnight fast (not eating for 10 hours)*

***SUBJECT SELECTION CRITERIA***

***Inclusion criteria:***

- *You are aged between 18-45 years*
- *You have body mass index (BMI) < 30 Kg/m^2^*

***Exclusion criteria:***

***Volunteers will be excluded if they have / are***

- *Chronic or metabolic disease (eg: type 2 diabetes, cardiovascular disease)*
- *Auto-immune disease (rheumatoid arthritis, type 1 diabetes, lupus)*
- *Liver disease and Anaemia*
- *On medications for control of blood glucose levels*
- *Neurological diseases or seizures*
- *On regular dietary supplements known to influence blood glucose level*
- *Pregnant, planning to become pregnant or breastfeeding*
- *Difficulties in providing blood sample*
- *Difficulties in providing informed consent*
- *Sensitivity/ intolerance to curcumin and/or fish oil, and food (dairy products, wheat, cow’s milk, protein and gluten)*

## Blood sample

### Blood sample collection

Blood samples will be collected according to standard operating procedures at the NRG

Blood sample storage and shipment

All blood samples will be destroyed after analysis.

### Blood sample assessment

All analytical procedures required for this proposal are routinely performed in the co-investigator’s laboratory and by a commercial pathology service provider.

Blood analysis for blood glucose, insulin and HbA1c will be analyzed by the commercial pathology service provider (HNEAPS).

Plasma curcumin and n-3PUFA concentrations will be analyzed by the Nutraceuticals Research Group Laboratory at the University of Newcastle.

# Safety Evaluation

## Potential risks

Investigators are responsible for monitoring the safety of participants who have entered this study and Professor Manohar Garg will be alerted to any event that seems unusual, even if this event seems to be an unanticipated benefit to the participant.

A health, diet and lifestyle questionnaire will be completed by the participants along with a signed informed consent, which will include medical conditions, current medication and dietary supplement use. The investigator will go through each response on the questionnaire with the participant at the start of the screening visit. Any adverse events or serious adverse events (see 11.1 for definitions) during the study will be reported to Professor Manohar Garg and to the HREC.

Blood test – Blood collection will be terminated if the participant experiences any adverse reactions or wishes to terminate the procedure. A slight sting may be experienced by participants during venepuncture. A blood sample of 20 mL (1 large tablespoon) will be collected which is less than 1% total blood volume and this represents no hemodynamic risk to participants. Nonetheless, there is also a risk of minor bruising. Participants will be advised to avoid lifting anything heavy for 24 hr after blood collection.

Investigational product –

The combination of Curcumin and n-3PUFA has yet to be tested in the humans. However, according to the scientific literature there no reports of any serious adverse events in humans regarding these nutraceuticals, as these are ingredients were the daily consumptions of humans (turmeric and fish) (Chuengsamarn, Rattanamongkolgul et al. 2012, Maki, Orloff et al. 2013). Minor side effects like gastric disturbances, nausea were reported at very large doses of 3.6g/ day for curcumin were reported in few studies (Sharma, Euden et al. 2004)

# Statistical Plan

## Sample Size Determination

Sample size determination

Seventeen participants in a 2x2 factorial study design will give 80% power to detect a 0.5 units drop in HbA1c at alpha = 0.05. To allow for dropouts we will recruit 4x20 = 80 participants according to the inclusion criteria.

## Statistical Methods

### 10.2.1Baseline data

Baseline measures will be used as covariates. Gender and BMI other potentially confounding variables such as weight, duration of pre-diabetes levels may be added as covariates if they are significantly correlated with the outcome measures.

### 10.2.2Treatment effects

All the data relating the significant effects of Curcumin and/or n-3PUFA are expressed as mean ± SEM. The effect of interventions on blood glucose levels and HbA1C between groups will be estimated by using two-way ANOVA with post hoc comparisons (Tukey’s honestly significant difference).

Significance (P-value set at 0.05) indicates the changes from the baseline values. Changes from the baselines will be determined using nonparametric analysis (Wilcoxon’s signed ranked test). This statistical analysis helps in determining whether there will be a significant main effect for each independent variable by testing for between subject effects. The statistical analysis by this method will be performed to evaluate the synergistic effects between Curcumin and n-3PUFA

## Subject Population(s) for Analysis

Data collected from participants who completed all study time points will be used for analysis.

# Safety and Adverse Events

## Definitions

**Unanticipated Problems Involving Risk to Subjects or Others**

Any incident, experience, or outcome that meets all of the following criteria:

- Unexpected in nature, severity, or frequency (i.e. not described in study-related documents such as the IRB-approved protocol or consent form, the investigators brochure, etc)
- Related or possibly related to participation in the research (i.e. possibly related means there is a reasonable possibility that the incident experience, or outcome may have been caused by the procedures involved in the research)
- Suggests that the research places subjects or others at greater risk of harm (including physical, psychological, economic, or social harm).

**Adverse Event**

An ***adverse event*** (AE) is any symptom, sign, illness or experience that develops or worsens in severity during the course of the study. Intercurrent illnesses or injuries should be regarded as adverse events. Abnormal results of diagnostic procedures are considered to be adverse events if the abnormality:

- results in study withdrawal
- is associated with a serious adverse event
- is associated with clinical signs or symptoms
- leads to additional treatment or to further diagnostic tests
- is considered by the investigator to be of clinical significance

**Serious Adverse Event**

Adverse events are classified as serious or non-serious. A ***serious adverse event*** (SAE) is any AE that is:

- fatal
- life-threatening
- requires or prolongs hospital stay
- results in persistent or significant disability or incapacity
- a congenital anomaly or birth defect
- an important medical event

Important medical events are those that may not be immediately life threatening, but are clearly of major clinical significance. They may jeopardize the subject, and may require intervention to prevent one of the other serious outcomes noted above. For example, drug overdose or abuse, a seizure that did not result in in-patient hospitalization, or intensive treatment of bronchospasm in an emergency department would typically be considered serious.

All adverse events that do not meet any of the criteria for serious should be regarded as ***non-serious adverse events***.

The study coordinator will inform the principal investigator of all AEs and SAEs, who will then follow procedures for unblinding as necessary and notify the relevant bodies. Depending on the nature of AE or SAE, it may be necessary for treatment to cease and/or for the participant to be withdrawn from the study.

All SAE that may be related or unrelated to the investigational product will be documented in the SAE Form, Case Report Form and will be reported immediately to the Human Research Ethics Committee (HREC) and the sponsors according to the conditions set in the Materials Transfer Agreement and the Grant Agreement. Any unforeseen AE, or complaints from participants in the research, or about the research, will be documented in the AE Form and Case Report Form and will be reported to the HREC as soon as possible.

All adverse events must be documented and followed up until the AE outcome has been established or the condition is stabilized, even after the subject has completed his/her study treatment.

Should there be a serious adverse event deemed related to the investigative treatment that increases the risks to participants, the study will be stopped, an investigation conducted, and findings generated before the study is resumed.

All AEs and SAEs must also be recorded on the appropriate page of the case report form (adverse event page and SAE form).

# Data Handling and Record Keeping

## Confidentiality

Confidentiality of participants will be maintained. Participant identity will be limited to authorized staff working on this study. However, in the event of an official audit and inspection, the authorized auditor will have access to the source documents for source data verification at the research site only.

Participants will be assigned a unique participant identification code. All data collected for the purposes of this study will be kept a separate folder and participants will not be identified from these folders. Prior to data archiving, the first page of the Health, Diet and Lifestyle Questionnaire containing participant’s contact details will be removed from the rest of the document and destroyed according to the University’s secure data disposal procedures.

With the participant’s consent, the study investigators may contact their nominated GP regarding their study participation if necessary. However, all participants are advised to discussion their involvement with their GP personally.

All individual data sets will be retained by the study investigators. Individual volunteers will not be identified in any reports arising from the study.

All data supplied to the sponsor will also be de-identified.

However, for the reporting of AEs and SAEs, participant’s characteristics including but not limited to age, anthropometric measurements, gender will be shared with sponsor. Participant’s name and contact details will not be released.

At the conclusion of the study, participants will be given an overall group summary of findings. Individual results of the outcome measures will not be released to the participants. A copy of the blood test results obtained at baseline visit may be given to participant if requested.

## Records Retention

Any information collected by the investigators which might identify participant will be stored securely and only assessed by the investigators and the authorized auditor. When a participant expresses initial interest in the study, a numeric identification code will be assigned to the volunteer. This numeric identification code will be used in all hard copies and electronic records of the data collected from each volunteer. The Health, Diet and Lifestyle questionnaire which participants will return by post, or via email, will bear the numeric identification code and contact details. The blood request form will bear the participant’s name and date of birth for identification purposes at the pathology collection centre. The form will not bear the participant’s study ID. During statistical data analysis the database will be stored in a password protected computer file on a computer that is kept in a locked room. All data for the study will be retained on file by the principal investigators at the University of Newcastle, in a locked data storage site for a period of 20 years. Electronic files are secured by password only known to the investigators of this study. All records including electronic files will be destroyed and deleted after 20 years. No blood samples will be stored. Blood samples will be destroyed once data for data have been analysed.

# Study Monitoring, Auditing, and Inspecting

## Study Monitoring Plan

The investigator will allocate adequate time for such monitoring activities. The Investigator will also ensure that the monitor or other compliance or quality assurance reviewer is given access to all the above noted study-related documents and study related facilities (e.g. pharmacy, diagnostic laboratory, etc.), and has adequate space to conduct the monitoring visit.

## Auditing and Inspecting

The investigator will permit study-related monitoring, audits, and inspections by the EC/IRB, the sponsor, government regulatory bodies, and University compliance and quality assurance groups of all study related documents (e.g. source documents, regulatory documents, data collection instruments, study data etc.). The investigator will ensure the capability for inspections of applicable study-related facilities (e.g. pharmacy, diagnostic laboratory, etc.).

Participation as an investigator in this study implies acceptance of potential inspection by government regulatory authorities and applicable University compliance and quality assurance offices.

# Ethical Considerations

Clinical research involving human subjects at CNRC has to be conducted in the respect of the following codes:

- The “Declaration of Helsinki” and its subsequent amendments;
- The Principles of Good Clinical Practice (GCP)
- The applicable laws and regulatory requirements governing the conduct of biomedical research projects involving human subjects.

GCP is a standard for the design, conduct, performance, monitoring, auditing, recording, analyses, and reporting of biomedical research that provides assurance that the data and reported results are credible and accurate, and that the rights, integrity, and confidentiality of subjects participating in biomedical research projects are protected.

All subjects for this study will be provided a consent form describing this study and providing sufficient information for subjects to make an informed decision about their participation in this study. This consent form will be submitted with the protocol for review and approval by the HREC for the study. The formal consent of a subject, using the EC/IRB-approved consent form, must be obtained before that subject undergoes any study procedure. The consent form must be signed by the subject or legally acceptable surrogate, and the investigator-designated research professional obtaining the consent.

# Investigator Responsibilities

It is the responsibility of the investigator(s) to conduct the study according to the protocol and to ensure that they have the subjects available to conduct the study within the period defined in the study protocol.

## Ethics committee approval

It is the responsibility of the investigator(s) to submit a copy of the protocol, a detailed subject information sheet and consent form (and any other required documents) to an HREC in order to obtain independent approval to conduct the study. HREC approval must be obtained before the study is started. The approval of the HREC must be sent in writing, to the investigator(s); The clinical trial will be registered with the Australia New Zealand Clinical Trials Registry prior to study commencement.

## Subject information and obtaining consent

It is the responsibility of the investigator(s) to obtain informed consent from each subject participating in the study, after explanation of the aims, methods, benefits and potential hazards of the study. The consent must be obtained before any study-specific procedures are performed. Only subjects who are able to give legal consent will be entered into the study. It must be made completely and unambiguously clear to each subject that they are free to refuse to participate in the study, or that they can withdraw their consent at any time and for any reason, without incurring any penalty or withholding of treatment on the part of the investigator. Signed informed consent must be kept on file by the investigator(s), and documented in the case report form (CRF) and the subject's medical records.

## Withdrawals of subjects

The investigator(s) have the right to withdraw a subject for any reason which is in the best interests of the subject, including intercurrent illness and adverse events). Whenever a subject is withdrawn from the study, for whatever reason, the subject may choose not to state the reason for withdrawal. Withdrawals due to non-attendance must be followed up to obtain the reason for non-attendance. Withdrawals due to intercurrent illness or adverse events must be fully documented in the case report form, with supplementary information where available and/or appropriate.

## Subject confidentiality

The investigator(s) will keep a separate Subject Identification Log which matches identifying codes with the subject's names and maintained by the investigator in strict confidence.

## Training of staff

The investigator will ensure that appropriate training relevant to the study is given to all of the study staff and that any new information of relevance to the performance of the study is forwarded to the staff involved.

## Protocol amendments

Any Amendments in the clinical protocol will be submitted to the HREC. These procedures must be fulfilled before any modification is put into effect.

## Case Report Form

It is the responsibility of the investigator(s) to ensure the completeness and accuracy of case report forms (CRF). One CRF must exist for each subject participating in the study. The case report form must be completed legibly, using a black ball point pen. Erroneous values and/or text must not be obliterated. Instead, the error must be crossed out with a single line, the correct value/text added, and the correction signed, initialized and dated by the investigator(s).

## Source document and source data verification

According to the standards of the data protection law, all data obtained in the course of a human study must be treated with discretion in order to guarantee the rights of the subject's privacy. The investigator should agree to allow the auditor/inspector to have access to any or all the study materials needed for proper review of the study progress.

# Study Finances

## Subject Stipends or Payments

Participants who complete the study will each receive $40 as a compensation for their time and expenses. Pro-rata payment will be made to participants who withdraw prior to study completion, $20 per initial and final visit. There will be no reimbursement for participants who did not attend the NRG for the screening/baseline visit or did not qualify for the study.

# Study Report and Publication Plan

A final study report and financial report will be written according to applicable ICH E3 guidelines and sent to the sponsors.

If successful, there is potential to submit a number of manuscripts for publications in high-ranked journals in the field of clinical nutrition or metabolic health.

Authorships on manuscripts will be followed according to the National Health and Medical Research Council and the Australia Research Council’s Australian Code for the Responsible Conduct of Research Section 5 on Authorship.

# Archiving

Archiving of all study materials will commence once all manuscripts have been published. The study investigators at the University of Newcastle must archive the protocol, documentation, approvals and all other essential documents related to the study, including certificates that satisfactory audit and inspection procedures have been carried out. Copies of all human study material must be archived for a period of at least 20 years (or more when legally required). All documents must be archived in a secure place and treated as confidential material.

# References

1. Grundy SM. Pre-Diabetes, Metabolic Syndrome, and Cardiovascular Risk. Journal of the American College of Cardiology. 2012;59(7):635-43.

2. Tabák AG, Herder C, Rathmann W, Brunner EJ, Kivimäki M. Prediabetes: a high-risk state for diabetes development. The Lancet.379(9833):2279-90.

3. Abdul-Ghani MA, Tripathy D, DeFronzo RA. Contributions of beta-cell dysfunction and insulin resistance to the pathogenesis of impaired glucose tolerance and impaired fasting glucose. Diabetes Care. 2006;29(5):1130-9.

4. Dunmore SJ, Brown JE. The role of adipokines in beta-cell failure of type 2 diabetes. The Journal of endocrinology. 2013;216(1):T37-45.

5. Agrawal NK, Kant S. Targeting inflammation in diabetes: Newer therapeutic options. World J Diabetes. 2014;5(5):697-710.

6. Aggarwal BB. Targeting inflammation-induced obesity and metabolic diseases by curcumin and other nutraceuticals. Annual review of nutrition. 2010;30:173-99.

7. Darnay BG, Singh S, Chaturvedi MM, Aggarwal BB. The p60 Tumor Necrosis Factor (TNF) Receptor-associated Kinase (TRAK) Binds Residues 344–397 within the Cytoplasmic Domain Involved in TNF Signaling. Journal of Biological Chemistry. 1995;270(25):14867-70.

8. Sharma RA, Euden SA, Platton SL, Cooke DN, Shafayat A, Hewitt HR, et al. Phase I clinical trial of oral curcumin: biomarkers of systemic activity and compliance. Clin Cancer Res. 2004;10(20):6847-54.

9. Lombardo YB, Chicco AG. Effects of dietary polyunsaturated n-3 fatty acids on dyslipidemia and insulin resistance in rodents and humans. A review. The Journal of nutritional biochemistry. 2006;17(1):1-13.

10. Horakova O, Medrikova D, van Schothorst EM, Bunschoten A, Flachs P, Kus V, et al. Preservation of metabolic flexibility in skeletal muscle by a combined use of n-3 PUFA and rosiglitazone in dietary obese mice. PLoS One. 2012;7(8):e43764.

11. Flachs P, Horakova O, Brauner P, Rossmeisl M, Pecina P, Franssen-van Hal N, et al. Polyunsaturated fatty acids of marine origin upregulate mitochondrial biogenesis and induce beta-oxidation in white fat. Diabetologia. 2005;48(11):2365-75.

12. Ramel A, Martinez A, Kiely M, Morais G, Bandarra NM, Thorsdottir I. Beneficial effects of long-chain n-3 fatty acids included in an energy-restricted diet on insulin resistance in overweight and obese European young adults. Diabetologia. 2008;51(7):1261-8.

13. Lopez-Alarcon M, Martinez-Coronado A, Velarde-Castro O, Rendon-Macias E, Fernandez J. Supplementation of n3 long-chain polyunsaturated fatty acid synergistically decreases insulin resistance with weight loss of obese prepubertal and pubertal children. Archives of medical research. 2011;42(6):502-8.

14. Dangardt F, Chen Y, Gronowitz E, Dahlgren J, Friberg P, Strandvik B. High physiological omega-3 Fatty Acid supplementation affects muscle Fatty Acid composition and glucose and insulin homeostasis in obese adolescents. Journal of nutrition and metabolism. 2012;2012:395757.

15. Liu A, Lou H, Zhao L, Fan P. Validated LC/MS/MS assay for curcumin and tetrahydrocurcumin in rat plasma and application to pharmacokinetic study of phospholipid complex of curcumin. J Pharm Biomed Anal. 2006;40(3):720-7.

16. Belcaro G, Cesarone MR, Dugall M, Pellegrini L, Ledda A, Grossi MG, et al. Efficacy and safety of Meriva(R), a curcumin-phosphatidylcholine complex, during extended administration in osteoarthritis patients. Alternative medicine review : a journal of clinical therapeutic. 2010;15(4):337-44.

17. Maki KC, Orloff DG, Nicholls SJ, Dunbar RL, Roth EM, Curcio D, et al. A highly bioavailable omega-3 free fatty acid formulation improves the cardiovascular risk profile in high-risk, statin-treated patients with residual hypertriglyceridemia (the ESPRIT trial). Clinical therapeutics. 2013;35(9):1400-11 e1-3.
